# Transcatheter Aortic Valve Migration During Vascular Access Closure

**DOI:** 10.7759/cureus.47067

**Published:** 2023-10-15

**Authors:** Jeet J Mehta, Christopher Scoma, Bibhu D Mohanty

**Affiliations:** 1 Cardiovascular Disease, University of South Florida Morsani College of Medicine, Tampa, USA

**Keywords:** tavr, perclose proglide, iatrogenic complication, prosthetic valve migration, transcatheter aortic valve replacement, aortic stenosis (as)

## Abstract

We present a novel complication of transcatheter aortic valve replacement (TAVR) involving prosthetic migration due to entanglement by a standard guidewire during non-large bore vascular access closure, followed by successful bail-out using a second transcatheter prosthesis. To our knowledge, this mechanism of prosthesis migration has not been previously described.

## Introduction

Periprocedural transcatheter aortic valve replacement (TAVR) device migration is rare, with event rates up to 0.9% [1]. Rates have decreased over time with operator experience; however, migration is associated with a threefold increase in mortality and accounts for >40% of TAVR-related emergent surgeries [1,2]. Risk factors include self-expanding prostheses and bicuspid valves [1]. There are multiple anatomic and procedural causes of device migration, including improper deployment location, undersized prostheses, ventricular hypertrophy, rapid-pacing failure, and equipment failures such as incomplete inflation or manufacturing defects [3]. We present a case involving TAVR migration with a successful bail-out utilizing a second prosthesis.

## Case presentation

A 69-year-old woman with a history of hypertension, diabetes mellitus, lymphoma treated with chemotherapy and radiation, and a distant history of stroke with mild residual left-sided weakness presented for TAVR to treat severe aortic stenosis (AS). Her pre-intervention echocardiogram demonstrated severe calcific aortic valve stenosis with a calculated aortic valve area of 0.9 cm^2^. She underwent uncomplicated deployment of a 26 mm Evolut Pro (Medtronic) transcatheter heart valve (THV) at an ideal annular depth (3 mm) using the right femoral approach, moderate conscious sedation, and the cusp-overlap technique without re-positioning. Intra-procedural transthoracic echocardiography (TTE) demonstrated excellent valve function without regurgitation or pericardial effusion, and normal left ventricle (LV) function. The patient remained in sinus rhythm without QRS prolongation. The delivery system and guidewire were removed sequentially under fluoroscopy, and the right-sided valve-access site was closed by the preclosure method using two ProGlide™ (Abbott) devices. The temporary pacing wire and pigtail catheter, placed in the left femoral vein and artery, respectively, were removed. The patient remained communicative and was breathing comfortably; she had normal vital signs, intact distal pulses, and no femoral access site oozing or hematoma.

A standard 0.035-inch J-tip guidewire was advanced from the left femoral 6 Fr arterial access site in preparation for ProGlide™ suture mediated closure. The sheath was removed and the ProGlide™ device was inserted; however, an attempt to remove the wire was met with firm resistance; the wire seemed to be fixed despite several withdrawal attempts. Using fluoroscopy, it was discovered that the wire tip had become knotted around the superior THV cells, causing aortic migration out of the annulus due to direct force applied on the knotted wire.

The differential diagnosis of TAVR device migration includes suboptimal deployment position, use of undersized prosthetics, failure of rapid pacing, equipment malfunction (including delivery equipment interaction), and vascular damage. The chance of a standard J-wire self-knotting a prosthesis so tightly as to pull it back was not even conceived as a possibility.

**Figure 1:**
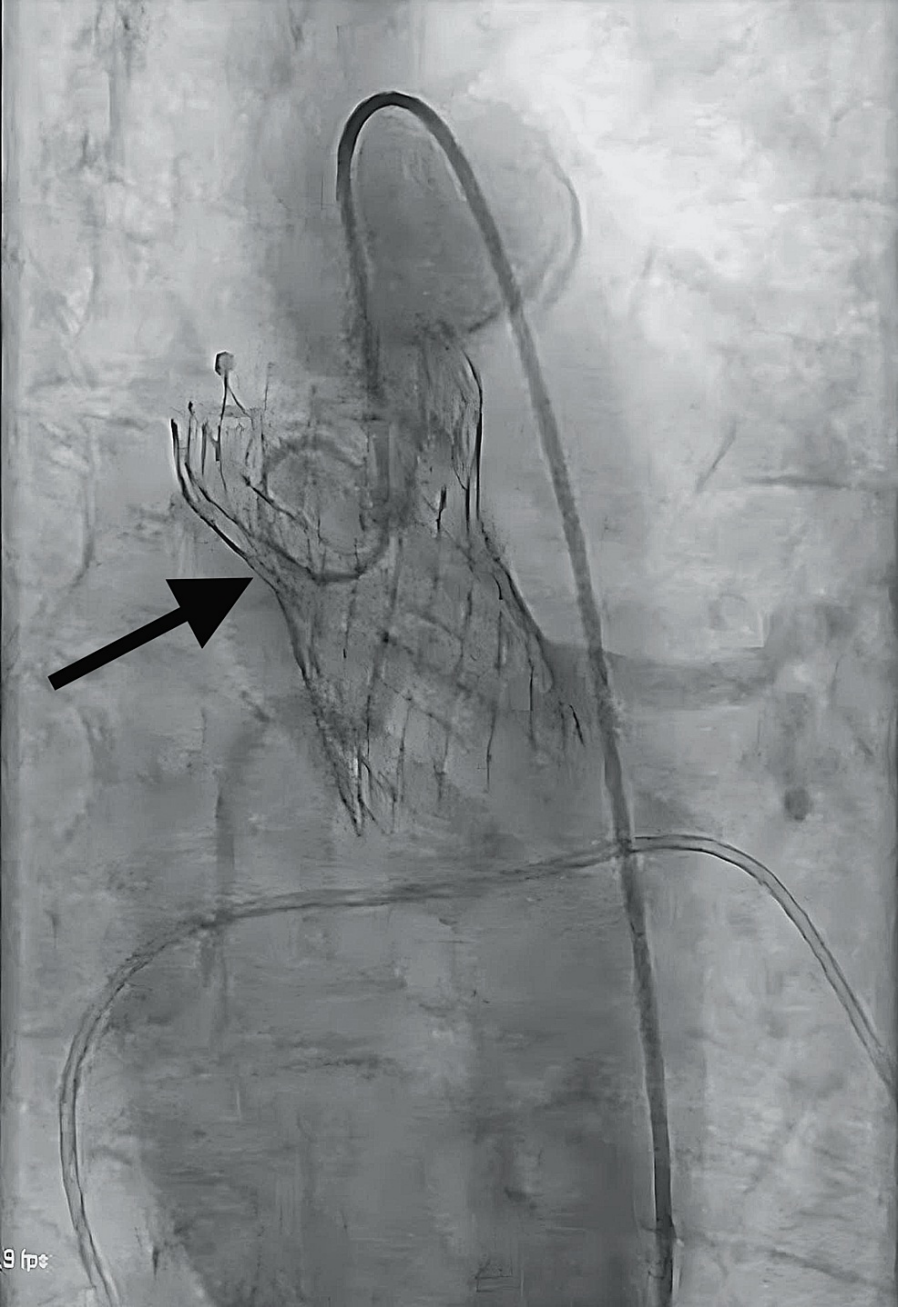
Cine-fluoroscopy of the migrated transcatheter heart valve (THV). Fluoroscopy shows superior migration of the THV into the ascending aorta with patent left and right coronary arteries.

Immediate angiography by pigtail catheter reinsertion showed the distal portion of the valve in the sinus of Valsalva, with intact coronary flow (Figure 1).

Electrocardiography showed normal sinus rhythm without ST-segment abnormalities. TTE showed aortic displacement of the THV without pericardial effusion or a change in LV function.

Under fluoroscopy, the knotted wire was disentangled from the valve. Given unobstructed coronary flow and maintaining TTE monitoring, immediate heart-team discussion was convened. Based on the ease of the initial deployment, the decision was made to pursue percutaneous management using a second Evolut Pro THV, contingent upon maintaining the distal end of the first valve above both coronary ostia and anticipating that the outflow portion of the second THV would anchor the displaced valve in the tubular ascending aorta.

**Figure 2:**
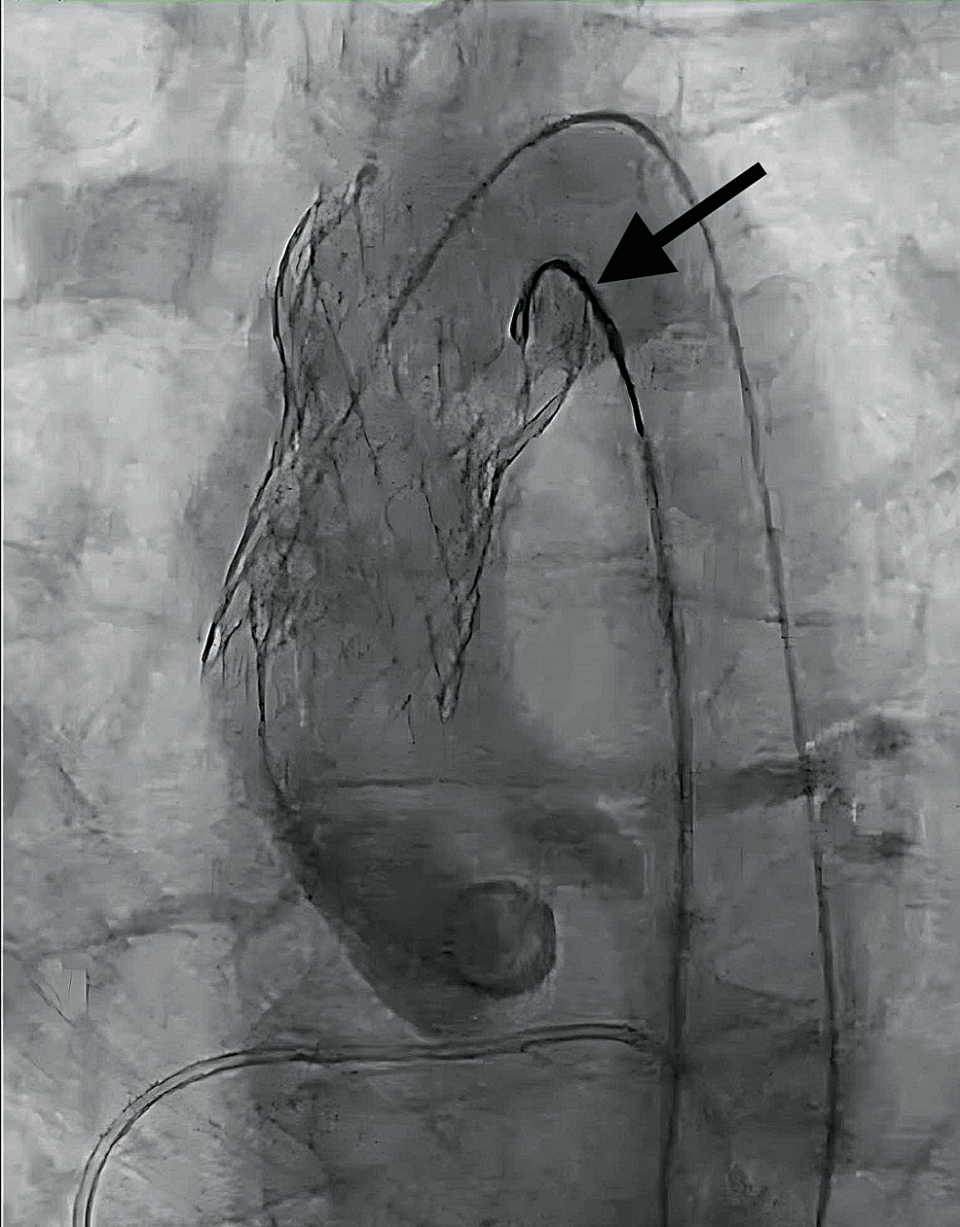
Transcatheter heart valve (THV) snaring. The THV was snared with a 20 mm Gooseneck snare and pulled back into the ascending aorta superior to the coronary ostia.

A new 9 Fr sheath was placed in the right femoral artery, through which a 4 Fr pigtail catheter and a 5 Fr JR-4 catheter housing a 20 mm Gooseneck snare were advanced to the level of the initial valve. The preexisting access site on the left was upsized to permit the second valve advancement. The displaced THV was snared and gently pulled back into the ascending aorta, distal to the coronary ostia but proximal to the brachiocephalic ostium (Figure 2).

**Figure 3:**
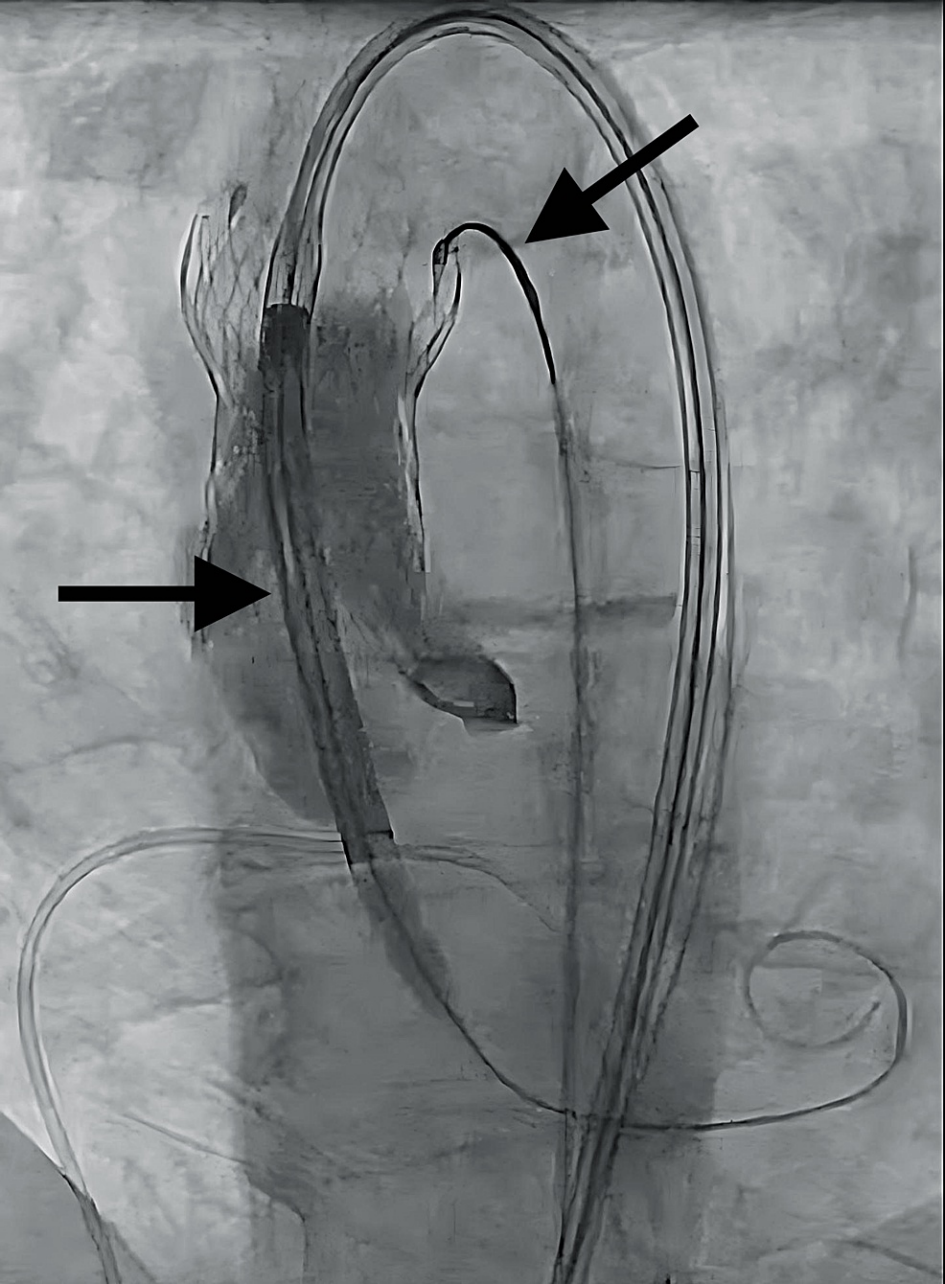
Second transcatheter heart valve (THV) delivery. Back-tension was maintained on the original THV as a new prosthesis was delivered into the aortic root.

Back-tension was maintained while ventricular access was prepared for the advancement of the second THV (Figure 3). 

**Figure 4:**
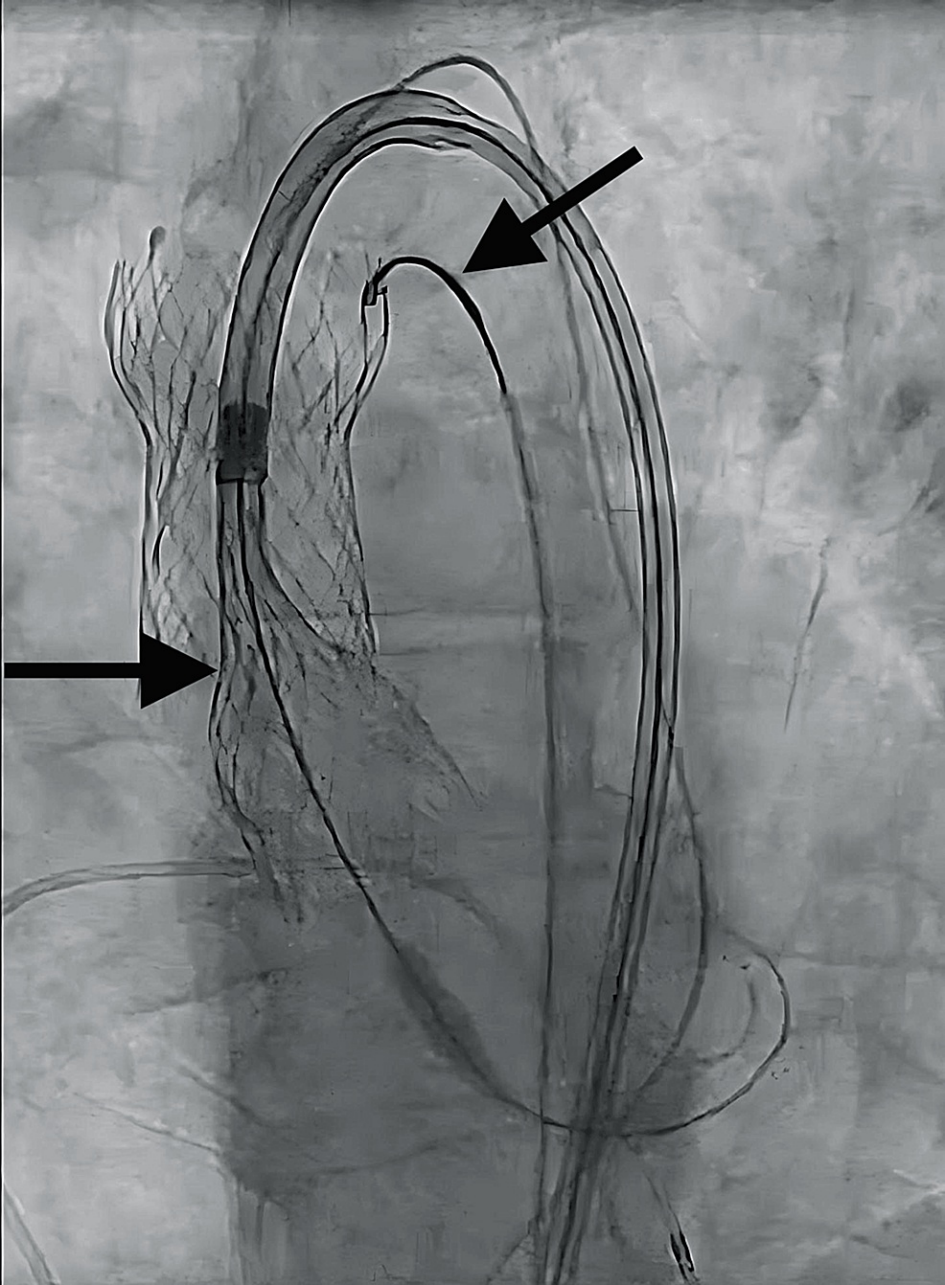
Second transcatheter heart valve (THV) deployment. The second THV self-expanded into the appropriate location while tacking down the leaflets of the original THV.

**Figure 5:**
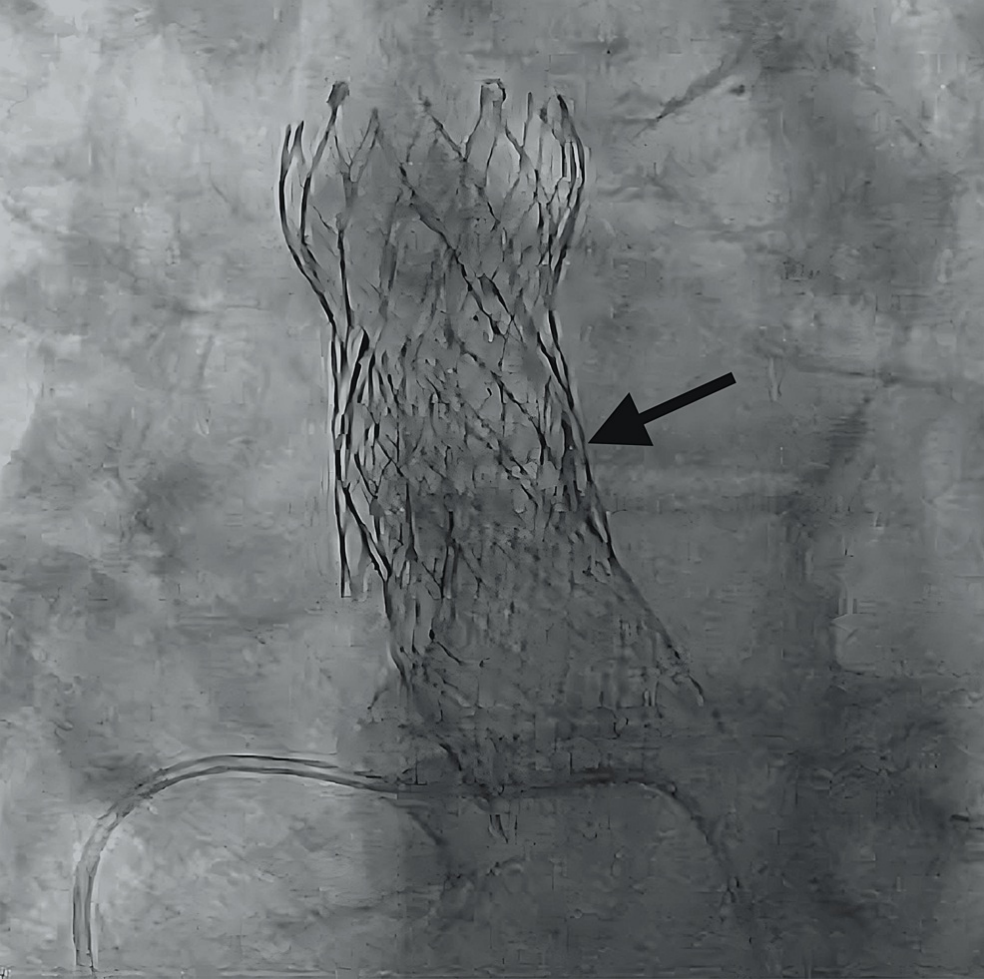
Final dual transcatheter heart valve (THV) location. The second THV deployed appropriately in the aortic root.

Tension was briefly relaxed to minimize interaction and aortic wall stress as the second prosthesis passed through the first. After passage, back tension was reapplied ensuring the displaced valve remained above the coronary ostia. The new THV was deployed in the annulus, with its superior portion anchoring the displaced valve in the ascending aorta (Figures 4, 5). 

**Figure 6:**
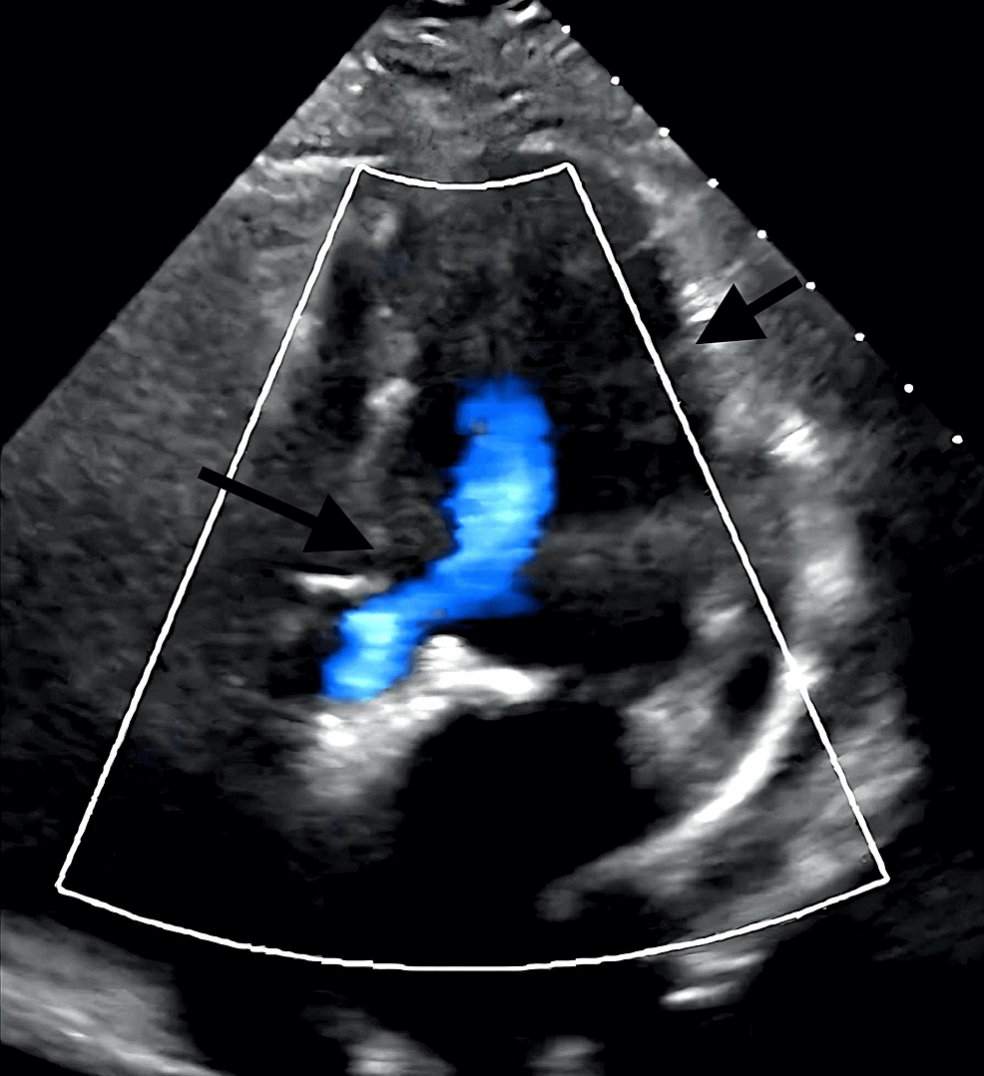
TTE color Doppler after TAVR without evidence of pericardial effusion. There is no post-deployment regurgitation by color Doppler assessment. TTE, transthoracic echocardiography; TAVR, transcatheter aortic valve replacement.

**Figure 7:**
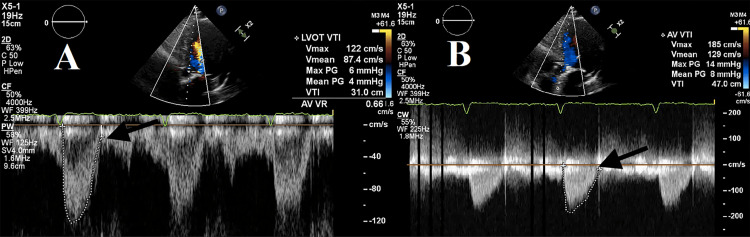
TTE spectral Doppler after TAVR of the LVOT (A) and aortic valve (B). Spectral Doppler tracings demonstrate trans-prosthetic peak velocity and dimensionless index within the normal range for valve type (1.85 m/s and 0.66, respectively). TTE, transthoracic echocardiography; TAVR, transcatheter aortic valve replacement; LVOT, left ventricular outflow tract.

TTE demonstrated normal prosthetic function (mean gradient 8 mmHg) without regurgitation, effusion, or change in LV function (Figure 6, 7A, 7B). 

Angiography demonstrated patent coronary flow. All equipment was then removed and both femoral vessels were closed with ProGlide™ devices, cautiously avoiding THV interaction. The patient remained asymptomatic throughout the procedure and was monitored overnight and discharged home the following day.

## Discussion

The management of device migration depends on the type of prosthesis (balloon vs. self-expandable), post-migration location (intra-cavitary, aortic), coronary patency, and the presence of vascular damage. Chawla et al. offer a decision-making pathway for displaced devices based on their location relative to coronary ostia and arch vessels and the feasibility of annular redeployment. If there is coronary or arch vessel obstruction, an attempt at repositioning or retraction should be made using a bioptome, balloon, or snare device. If repositioning is unsuccessful, emergent surgery should be considered [4].

In our case, despite an uneventful initial deployment, unintended guidewire-prosthesis entanglement occurred during non-valve access closure, which went unrecognized until retraction inadvertently caused THV displacement. Due to the location (without coronary or arch vessel obstruction) and hemodynamic stability, percutaneous management was feasible. The first THV was snared for aortic anchoring, and a second THV was delivered with favorable function.

Our case highlights the possibility that a benign guidewire can become knotted within a THV frame after deployment. ProGlide™ suture-mediated vascular closure requires several steps to achieve hemostasis, including the advancement of the device over a standard guidewire. There are several complications contingent on vascular anatomy and appropriate device use; however, to our knowledge, guidewire entanglement leading to THV migration has not previously been described. This case underscores the importance of fluoroscopy and deliberate guidewire insertion free of a prosthetic device to avoid such a drastic consequence in an otherwise uneventful procedure.

Following the procedure, the patient was admitted once for pericarditis, which resolved with nonsteroidal anti-inflammatory agents and colchicine. Echocardiography showed unchanged trans-prosthetic gradients without regurgitation, effusion, or changes in LV function. At six months, she is well at home without further hospitalizations.

## Conclusions

We have described, for the first time, the displacement of a THV due to frame-entanglement with a standard guidewire. Successful percutaneous bail-out was achieved by snaring and manipulation of the THV with placement of a second overlapping THV yielding a favorable clinical outcome.
